# Synpolydactyly and HOXD13 polyalanine repeat: addition of 2 alanine residues is without clinical consequences

**DOI:** 10.1186/1471-2350-8-78

**Published:** 2007-12-11

**Authors:** Sajid Malik, KM Girisha, Muhammad Wajid, Akhilesh K Roy, Shubha R Phadke, Sayedul Haque, Wasim Ahmad, Manuela C Koch, Karl-Heinz Grzeschik

**Affiliations:** 1 Zentrum für Humangenetik, Philipps-Universität Marburg, Bahnhofstr. 7, 35037 Marburg, Germany; 2 Department of Biological Sciences, Quaid-I-Azam University, 45320 Islamabad, Pakistan; 3 Department of Medical Genetics, Sanjay Gandhi Postgraduate Institute of Medical Sciences, Lucknow-226014, Uttar Pradesh, India; 4 Division of Pediatric Surgery, Department of Surgery, Era's Lucknow Medical College, Hardoi Road, Sarfarazganj, Lucknow-226003, Uttar Pradesh, India

## Abstract

**Background:**

Type II syndactyly or synpolydactyly (SPD) is clinically very heterogeneous, and genetically three distinct SPD conditions are known and have been designated as SPD1, SPD2 and SPD3, respectively. SPD1 type is associated with expansion mutations in *HOXD13*, resulting in an addition of ≥ 7 alanine residues to the polyalanine repeat. It has been suggested that expansions ≤ 6 alanine residues go without medical attention, as no such expansion has ever been reported with the SPD1 phenotype.

**Methods:**

We describe a large Pakistani and an Indian family with SPD. We perform detailed clinical and molecular analyses to identify the genetic basis of this malformation.

**Results:**

We have identified four distinct clinical categories for the SPD1 phenotype observed in the affected subjects in both families. Next, we show that a milder foot phenotype, previously described as a separate entity, is in fact a part of the SPD1 phenotypic spectrum. Then, we demonstrate that the phenotype in both families segregates with an identical expansion mutation of 21 bp in *HOXD13*. Finally, we show that the HOXD13 polyalanine repeat is polymorphic, and the expansion of 2 alanine residues, evident in unaffected subjects of both families, is without clinical consequences.

**Conclusion:**

It is the first molecular evidence supporting the hypothesis that expansion of ≤ 6 alanine residues in the HOXD13 polyalanine repeat is not associated with the SPD1 phenotype.

## Background

Type II syndactyly or synpolydactyly (SPD) is characterized by webbing between 3/4 fingers and between 4/5 toes with partial or complete digit duplication within the syndactylous web. Three loci have been identified at chromosomes 2q31, 22q13.31 and 14q11.2-q12, and have been designated as SPD1, SPD2 and SPD3, respectively [1-3]. Unique expansion mutations in a poly alanine repeat (PolAR) of HOXD13 have been implicated in SPD1 families [4,5]. Interestingly, all mutations discovered in at least 31 SPD1 families reported so far, were ≤ 7 alanine residue expansions. This observation has lead to a suggestion that expansion mutations ≤ 6 alanine residues do not cause the SPD1 malformation [4,6]. However, no clinical evidence has emerged to support this hypothesis.

Within SPD1 families, there exists a broad spectrum of inter- and intra-familial phenotypic manifestations. Even though, for the phenotypes segregating in large families unique clinical categories have been proposed which remain mutually exclusive [7]. Here, we describe a large Pakistani and an Indian family with SPD1, identify four distinctive clinical categories of the phenotype in the affected subjects, and give evidence for a minor foot phenotype being a part of the SPD1 clinical spectrum. We observe identical 7 alanine expansion mutations in HOXD13 in the affected subjects in both families. We further show that two normal variants of PolAR are segregating in both families and that an expansion of two alanine residues in PolAR is not associated with the SPD1 malformation.

## Case study

### Pakistani family

**Figure 1:**
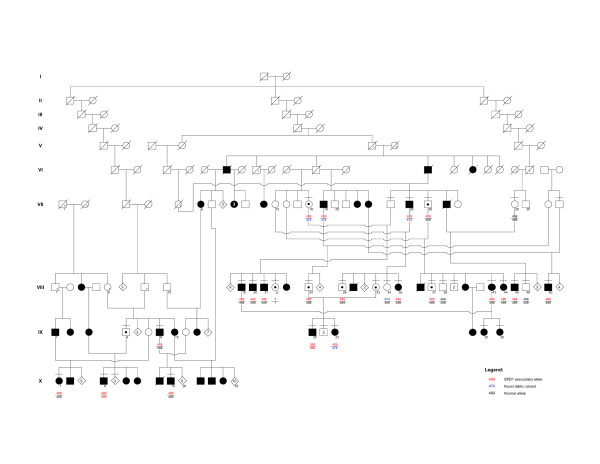
**Pedigree of the Pakistani kindred with SPD1**. Horizontal bars on the symbols denote individuals who were physically examined. Genotypes for the GCN-region of *HOXD13 *showing the repeat length (bp) are provided for the subjects available for molecular analysis. Non-penetrant subjects and obligate carriers are depicted with a dot within the symbols.

This large family originates from the Thal desert in Eastern Punjab, Pakistan. The pedigree was constructed by interviewing the tribal heads and the elders of the sub-families who had maintained remarkably extensive family records dating back several centuries. The family pedigree spans ten generations and ~250 years (Figure 1).

Forty-seven subjects (21 males, 26 females) were found to be affected, seven of which had been deceased (Figure 1). Seventeen affected and 21 normal subjects were physically examined. The clinical details were recorded according to the scheme described by Malik et al. [8]. Radiographs of three subjects (VIII-24, IX-20, IX-23), and peripheral blood samples of 26 available subjects (16 affected and 10 apparently normal) were obtained. All biological material was obtained after an informed consent according to Helsinki II declaration.

### Indian Family

A five generation Indian family was ascertained at the Era's Lucknow Medical College, Lucknow, India. A detailed pedigree was constructed and eight subjects were physically examined. Ten subjects (six males, four females) were found to be affected. After the informed consent, photographs and/or radiographs of five affected subjects (III-3, IV-7, V-3, V-4, V-6), and the blood samples of available subjects (IV-7, IV-8, V-3, V-4) were obtained.

## Methods

Genomic DNA was extracted according to standard methods. To test the candidate locus SPD1 at chromosome 2q31 for linkage with the phenotype segregating in the Pakistani family, microsatellite markers from this region were selected (D2S418, D2S1353, D2S2330, D2S2307, D2S2314, D2S138, and D2S1391). Genotyping and linkage analysis were performed as described before [9].

To screen *HOXD13 *for mutations, the coding sequences were PCR amplified with the primers described elsewhere [10], by employing the Advantage GC-Genomic PCR kit (CLONTECH, PaloAlto, CA, USA). The upstream and downstream regions flanking the first and last exon were amplified with the primer pairs: 5' → 3' (F1-agagggagagagggctagag, R1-ctacaacggcagaagaggac; F5-cttagaagccattcggttgt, R5-cagtaccttcagcctcctgt). Sequencing was performed on both strands with the BigDye Terminator Cycle Sequencing kit (Applied Biosystems, Foster City, CA, USA) with an automated ABI-377 genetic analyzer (Applied Biosystems). Trace chromatogram data were analyzed with Sequencher 4.7 software. For a robust screening of a polyalanine repeat expansion, one of the oligos from the primer pair exon1a [10], flanking the repeat, was fluorescently labelled (FAM), and the amplified PCR product was analyzed on a 6% denaturating polyacrylamide gel with the ABI-377 (TAMRA-500 as an internal control). Fragment analysis was performed using GeneScan (ver 3.1.2) and Genotyper (ver 2.0) softwares.

## Results

### Clinical report

**Figure 2:**
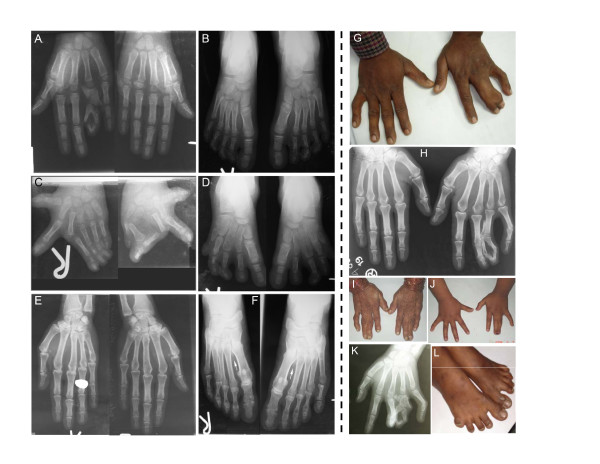
**SPD1 phenotype in the Asian families**. Radiographs and photographs showing the SPD1 phenotype in the Pakistani (A – F), and Indian (G – K) families. **A-B**, (subject IX-23); **C-D**, (IX-20); **E-F**, (VIII-24); **G-H**, (IV-7); **I**, (III-3); **J**, (V-6); **K-L**, (V-3). Phenotypic categories are given in Figure 3.

**Figure 3:**
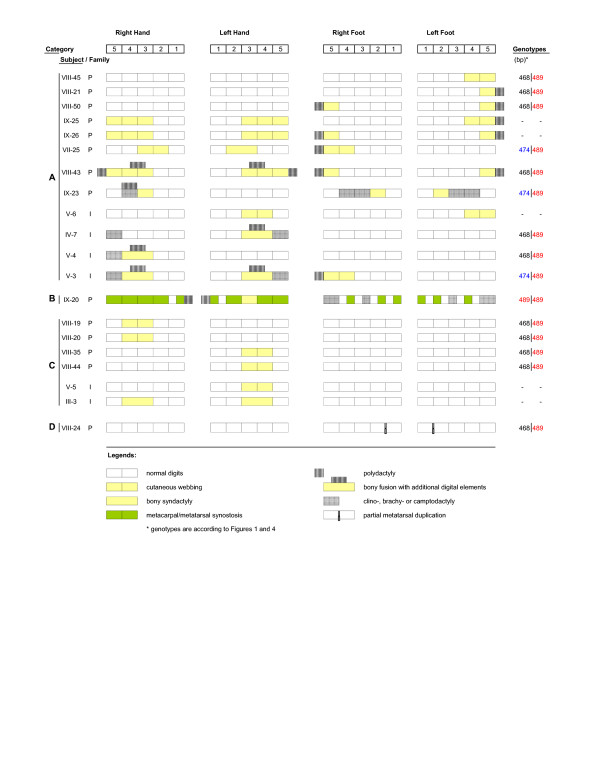
**Phenotypic categories (A, B, C, D) in the affected subjects of the Pakistani (P) and Indian families (I)**. SPD1 phenotypic variants observed in the Pakistani and Indian family subjects, are grouped into four distinct clinical categories (A, B, C, D), drawn according to the scheme described by Malik et al. [8]. Genotypes for the GCN-region of *HOXD13 *are also provided (see Figures 1; 4).

A broad spectrum of SPD phenotypes was evident in the affected subjects of both families. However, the phenotype could be divided into four unique clinical categories (Figures 2; 3):

1: Bony fusion of 3/4 fingers and/or post-axial synpolydactyly of toes, representing the typical SPD phenotype (Figures 2A, B, G, H, K, L; 3A);

2: Complete syndactyly of all digits of hands and feet, consistent with homozygous expression of the mutant allele (Figures 2C, D; 3B);

3: Isolated cutaneous fusion of 3/4 fingers, the feet being normal (Figures 2I, J; 3C);

4: Partially developed metatarsal between 1^st ^metatarsal spaces, a minor foot phenotype (Figures 2F; 3D).

### Molecular analyses

**Figure 4:**
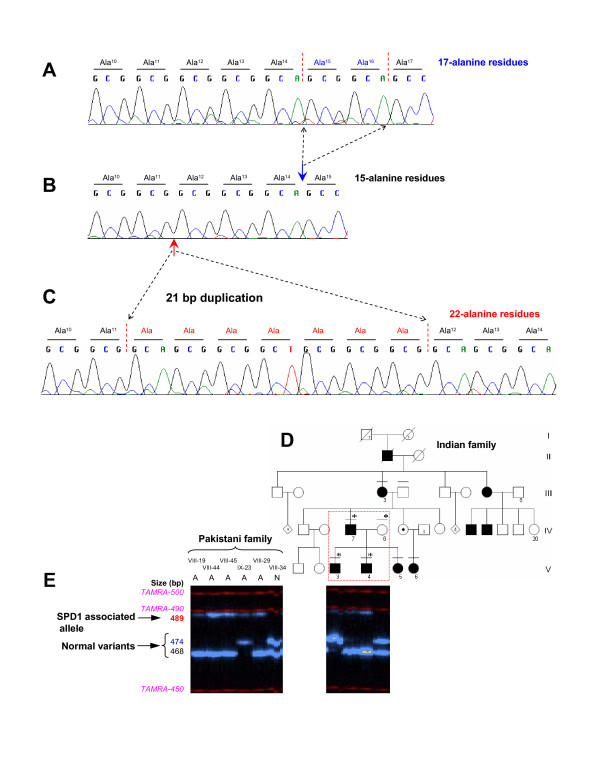
**Molecular analysis of HOXD13**. **A, B**: DNA electropherograms of the GCN-region of *HOXD13 *exon 1, depicting two normal repeat variants. **C**, SPD1 associated duplication of 21 bp in homozygous affected subject (IX-20) in the Pakistani kindred, results in an addition of seven alanine residues. **D**, Indian pedigree with SPD1; the part of the pedigree analyzed is highlighted in red. **E**, Normal repeat polymorphisms and the expansion mutation are evident by allele separation of the exon 1 PCR product flanking the GCN region.

Four microsatellite markers flanking the SPD1 candidate interval (D2S2330, D2S2307, D2S2314, and D2S2319) revealed significantly high LOD scores (*Z*_*max *_≥ 3), indicating a strong evidence of linkage between this locus and the phenotype segregating in the Pakistani family. A shared haplotype was present in all the affected subjects and in six of the apparently normal individuals, revealing a decreased penetrance (VII-18, VII-26, VIII-22, VIII-24, VIII-29, VIII-37) (Figure 1). However, a close examination of the radiograms of one of the apparently normal subjects (VIII-24) in fact established the presence of partially developed proximal second metatarsal within the first metatarsal spaces (Figures 2F; 3D). Subsequent mutation screening of *HOXD13 *showed a 21 bp expansion in the imperfect GCN triplet-repeat of exon 1, c.297_317dupGCAGCGGCGGCTGCGGCGGCG (Figure 4C). This mutation resulted in an addition of 7 alanine residues between the 11^th ^and 12^th ^alanine in PolAR. Subject IX-20 was found to be homozygous for this mutation, which explains his severe limb phenotype showing a bizarre arrangement of all digital elements of hands and hypo-/dys-plastic tarsals and metatarsals in feet (Figure 2C, D). Owing to the homozygous mutant status of this subject, his apparently unaffected mother (VIII-33) is an obligate carrier. However, her genetic status could not be established due to lack of a blood sample.

The mutation screening in the Indian Family also revealed an identical 21 bp expansion in the GCN triplet-repeat segregating with the phenotype (Figure 4E). Remarkably, in both Pakistani and Indian families we also observed two normal variants of triplet-repeat sequence (45 bp and 51 bp), resulting in 15 and 17 alanine residues, respectively, in PolAR of the HOXD13 protein (Figure 4A, E). The 6 bp repeat expansion was observed in five subjects in the Pakistani kindred (VII-18, VII-19, VII-25, VIII-34, IX-23) and in two subjects in the Indian family (IV-8, V-3), c.288_293dupGCGGCA. In both families, the expansion was segregating in affected as well as unaffected subjects, revealing that it is not linked to the malformation (Figure 4E). This 6 bp expansion was not observed in two additional large SPD1 families (data not shown), depicting the rarity of this variant. In exon 1, we found two synonymous sequence variants in the Pakistani family (c.180G → A; p.A60A, c.378C → T; p.G126G) and one in the Indian family (c.666G → A; p.Q222Q). No mutation was observed in exon 2 in either family.

## Discussion

Among all non-syndromic syndactyly types SPD is clinically and genetically the most heterogeneous malformation. However, in large families it is possible to group the affected subjects on the basis of clinical variants. For instance, four distinct clinical categories have been appreciated in a large Turkish family showing SPD1 [7]. Typical SPD phenotype and various milder variants were also observed in a panel of SPD1 families reported by Goodman et al. [4]. Likewise, in the Asian families we have identified four distinctive clinical categories (Figure 3A, B, C, D). At present there is no specific molecular explanation for the occurrence of discrete clinical variants in SPD1 families. However, variations in the genetic backgrounds reflecting the presence of modifier loci in different families or in different loops of the same family could be involved. It might also well be that stochasticity in *HOXD13 *dosage during development could results in the phenotypic variability [11].

A milder foot phenotype (partially developed metatarsals), observed in two families with less explicit SPD features has been advocated as a separate entity associated with deletion mutations in *HOXD13 *outside the PolAR [12]. In contrast, we suggest that this clinical variant is a part of the SPD1 phenotypic spectrum. First, this milder foot phenotype was observed in a large Turkish SPD1 family [7]. Then, a Caucasian family with this phenotype was reported in which typical SPD clinical features were present in only few affected subjects [11]. Finally, in the present Pakistani family we observe this milder foot phenotype as well. The frequent recurrence of this clinical variant with SPD1 leads us to conclude that it is in fact a milder phenotypic manifestation of SPD1 and not a separate entity in itself. Additionally, the presence of this variant calls for a more thorough phenotypic assessment in SPD1 families involving close radiological evaluation.

The PolAR expansion mutations in HOXD13 are believed to confer gain-of-function [4]. However, this gain-of-function is confined to expansion with a threshold total length of 22 alanine residues in PolAR (i.e., expansion of ≥ 7 alanines). Therefore, it has been argued that the expansions of ≤ 6 alanine residues remain without clinical consequences [6]. Our findings in two Asian families provide for the first time molecular evidence to reinforce this hypothesis, as we show that there is repeat polymorphism in PolAR and that an addition of 2 alanine residues in the PolAR is not associated with the SPD1 phenotype. The presence of identical repeat polymorphisms and mutations in two geographically different families is a rare finding. The observation in the Pakistani family shows that the mutation as well as the normal repeat polymorphism in PolAR are inherited meiotically stable over ~6 generations. Nonetheless, our data does not indicate the 2 alanine repeat expansion in itself has a modifier effect on SPD1 phenotype. The phenotype in subjects harbouring both a 7 alanine expansion and a 2 alanine expansion *in trans *is not remarkably distinct from the typical SPD phenotype. Additionally, it is worth mentioning that polyalanine contractions of two and four alanine residues have been described previously without obvious pathogenic effect [13,14], however, a 7 alanine residues contraction has been shown to be associated with brachydactyly/syndactyly phenotypes [15].

Since the proposal of purely clinical classification of non-syndromic syndactylies [16], the phenotypic spectrum of SPD has broadened extensively (reviewed in [17]). In certain instances, it overlaps with other well-characterized syndactyly types. For instance, isolated webbing of 3/4 fingers (MIM 185900) has been observed in the panel of SPD1 families reported by Goodman et al. [4], and is also present in the present two Asian families. Secondly, another separate entity, isolated 2/3 toes cutaneous syndactyly, has also been observed in SPD1 family subjects [4,13]. The phenotypic overlap of SPD1 with clinically and genetically distinct syndactylies calls for further investigations. It is quite likely that there is a crosstalk between *HOXD13 *and the type I syndactyly locus (or loci) in defining the limb phenotype. We are embarking upon further studies in order to understand this clinical and genetic heterogeneity observed in synpolydactyly families.

## Conclusion

Synpolydactyly is clinically and genetically very heterogeneous; however distinct non-overlapping clinical variants have been identified in families with synpolydactyly type I (SPD1). When a minor variant occurs as a predominant phenotype in a given family, it should not be considered as a separate entity. The expansion of two alanine residues in the polyalanine repeat of HOXD13 is not associated with an SPD phenotype.

## Competing interests

The author(s) declare that they have no competing interests.

## Authors' contributions

SM and MW recruited the Pakistani family subjects, SH and WA coordinated. KMG, AKR and SRP contributed the Indian family. KHG and MCK conceived and designed the study. SM conducted genotyping, linkage analysis and sequencing, and drafted the manuscript. All authors read and approved the manuscript.

## Pre-publication history

The pre-publication history for this paper can be accessed here:


